# Supplementary material for: Network-based near-scalp personalized brain stimulation targets
Source: Imaging Neurosci (Camb). 2026 May 21;4:IMAG.a.1222. doi: 10.1162/IMAG.a.1222 (PMC13195926; doi:10.1162/IMAG.a.1222)
Supplement: Supplementary Material [file IMAG.a.1222_supp.pdf]

Supplementary Material

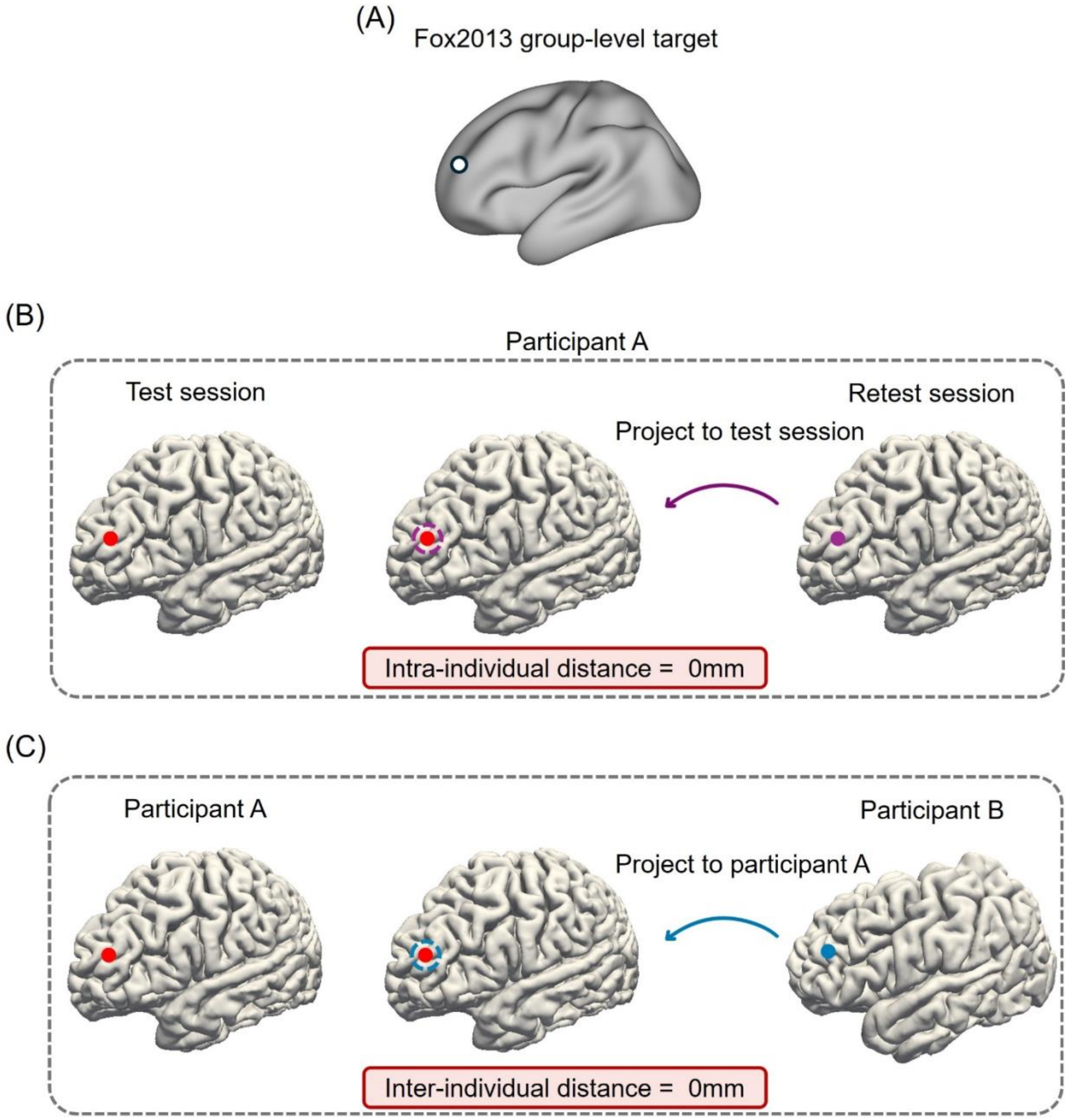

Figure S1. Illustration of a group-level target with perfect intra-individual reliability but no individual specificity. (A) A group-level target from MNI152 space (e.g., Fox et al., 2013). (B) The group-level target from MNI152 space (e.g., Fox et al., 2013) was anatomically projected to a participant's T1 in the "test" session and the participant's T1 in the "retest" session for neuromodulation. To compute test-retest reliability, the target in the "retest" session is projected to the "test" session (via MNI152 space) and compared with the target in the "test" session. In this scenario, the overlap between the transformed "retest" target and the "test" target

will be perfect and the intra-individual distance will be zero. (C) On the other hand, suppose the group-level target from MNI152 space (e.g., Fox et al., 2013) was anatomically projected to participant 1's T1 and ∂participant 2's T1 for neuromodulation. To compute inter-individual variability, the target in participant 2's T1 is projected to participant 1 T1 (via MNI152 space) and compared with participant 1's target. In this scenario, the overlap between the transformed participant 2's target and participant 1's target will be perfect and the inter-individual distance will also be zero. Thus, there is perfect intra-individual agreement, but no individual specificity.

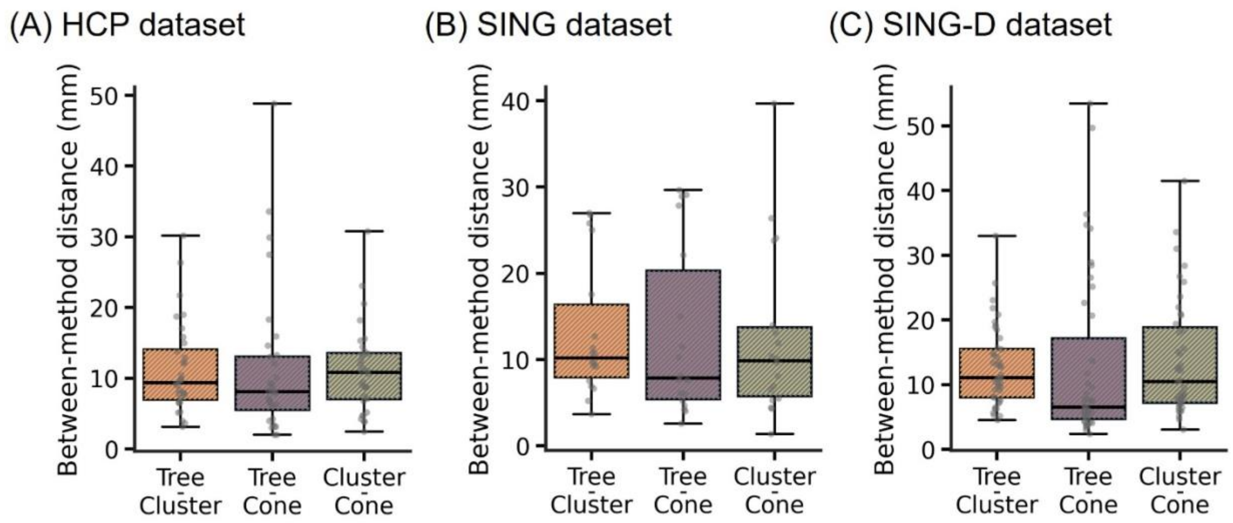

Figure S2. Pairwise Euclidean distances between depression targets derived from tree-based MS-HBM, cluster, and cone algorithms in (A) the HCP dataset, (B) the SING dataset, and (C) the SING-D dataset.

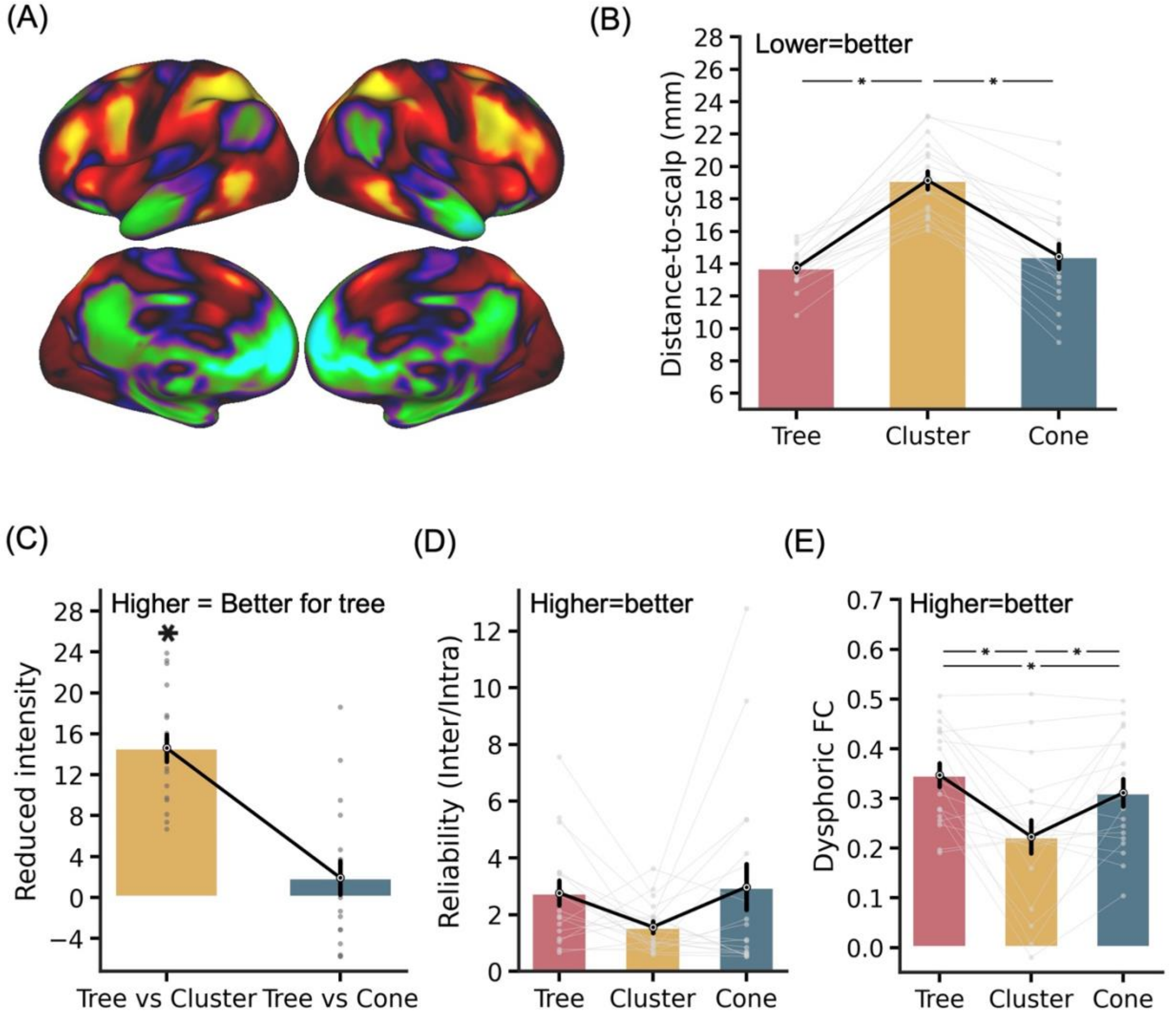

Figure S3. Personalized depression targets derived from tree-based MS-HBM using dysphoric circuit map. (A) Group-level dysphoric circuit map visualized on fsaverage6 surface. (B) Distance-to-scalp in the SING dataset. A smaller value indicates better performance. (C) Reduction in stimulation intensity in the SING dataset. Following the SAINT protocol (Cole et al., 2020), by using a linear adjustment in stimulation intensity based on distance-to-scalp and assuming 90% RMT dosage, a hypothetical reduction in stimulation intensity between tree-based MS-HBM and other approaches can be computed. (D) Reliability (ratio of inter-individual distance and intra-individual distance) in the multi-echo multi-band dataset. A higher value indicates better performance. (E) FC with the dysphoric circuit map in the SING dataset. A more positive value indicates better performance. * indicates statistical significance after multiple comparisons correction with FDR $q < 0.05$. We note that in all analyses, targets were derived in one session and then evaluated in another session.
